# The relationship between presence of meaning and life satisfaction among Chinese young adults: a moderated mediation model of perceived stress and hope

**DOI:** 10.3389/fpsyt.2025.1610440

**Published:** 2025-08-25

**Authors:** Yan Zhang, Bo Jiang, Tingting Lei, Yuhong Yan

**Affiliations:** College of Educational Science, Jiangsu Second Normal University, Nanjing, China

**Keywords:** presence of meaning, perceived stress, hope, life satisfaction, young adults

## Abstract

**Background:**

Research has revealed that presence of meaning in life may be a protective factor for life satisfaction among young adults in China. However, few studies have examined the underlying mechanisms that may mediate or moderate this association. This study aimed to test the mechanisms underlying the relationship between meaning in life and life satisfaction among young adults in China.

**Objective:**

This study aimed to examine the effects of presence of meaning in life on the life satisfaction among Chinese young adults, as well as the mediating role of perceived stress and moderating role of hope.

**Methods:**

A total of 909 young adults in China completed measures of presence of meaning, perceived stress, hope and life satisfaction. The participants were recruited from four large public universities located in Jiangsu Province in China. SPSS statistical analysis was used to evaluate the relationship between presence of meaning in life, perceived stress, hope and life satisfaction.

**Results:**

Presence of meaning in life was both positively associated with life satisfaction, controlling for grade and being from an urban or rural area (*β* = 0.46, *p* < 0.001). Perceived stress played a mediating role in the association between presence of meaning in life and life satisfaction (indirect effect = 0.08, *95%CI* = [0.05,0.13]). Furthermore, the interaction of presence of meaning in life and hope was positively related to life satisfaction (*β* = 0.14, *p* < 0.01) and the interaction of perceived stress and hope was positively related to life satisfaction (*β* = 0.11, *p* < 0.05). A high level of hope can enhance the link between presence of meaning in life and life satisfaction while reduce the link between perceived stress and life satisfaction.

**Conclusion:**

Presence of meaning in life was positively associated with life satisfaction among Chinese young adults. Furthermore, perceived stress played a mediating role in the association between presence of meaning in life and life satisfaction, and this mediating effect was moderated by hope.

## 1 Introduction

Well-being is the ultimate goal of human action. With the rise and development of positive psychology, psychological research on well-being has significantly progressed. As a measure of subjective well-being, life satisfaction refers to an individual’s cognitive evaluation of their overall quality of life (1). As a core concept of positive psychology, life satisfaction is considered an influential predictor of psychological health among young adults (2). Life satisfaction may serve as a composite health indicator because life dissatisfaction has been found to be a predictor of disease mortality and have a long-term effect on the risk of suicide (3). Meanwhile, the studies indicated that life satisfaction has a significant negative predictive effect on negative mental health outcomes (4) and Chinese young adults’ internet addiction (5). As a cohort shaped by China’s rapid urbanization, educational hyper-competition, and stringent family expectations, Chinese young adults face unique socio-developmental challenges. Therefore, it is necessary to investigate the mechanism of life satisfaction among Chinese young adults.

### 1.1 Presence of meaning in life and life satisfaction among Chinese young adults

Meaning in life has been an eternal topic for humanity, and its exploration accompanies different stages of the life process. Research on meaning in life began in the 1950s. Subsequently, in-depth research on meaning in life has been conducted in various fields of psychology, especially positive psychology (6). Based on the integration of several influential concepts of meaning in life, Steger, Frazier and Oishi (7) defined it as an individual’s perception of the essence of human beings and their existence, as well as those that they consider to be more important. Meaning in life includes two dimensions: presence of meaning and search for meaning. Presence of meaning refers to the degree to which an individual perceives their life as meaningful, emphasizing the outcome. Search for meaning refers to the degree to which an individual actively seeks meaning in life, emphasizing the process.

Constructing meaningful interpretations is an effective coping strategy for dealing with stressors (8). In this regard, meaning in life can not only enhance an individual’s immune system and promote physical health, but also significantly improve subjective well-being and life satisfaction (9–11). A meta-analysis based on Chinese samples showed a significant positive correlation between meaning in life and life satisfaction (12). The study demonstrated that presence of meaning in life and search for meaning represent distinct psychological constructs, necessitating separate investigations of their respective associations with life satisfaction (13). Previous studies have consistently found a positive relationship between presence of meaning in life and life satisfaction (12, 14). Furthermore, a study indicated that a large proportion of the stable variance in presence of meaning and life satisfaction is shared (9). However, results from previous studies on the relationship between search for meaning in life and life satisfaction are equivocal. In one study, search for meaning in life significantly negatively predicted life satisfaction (7), whereas in another, it significantly positively predicted life satisfaction (15). Therefore, this study focused on exploring the mechanisms underlying the relationships between presence of meaning in life and life satisfaction.

In particular, few studies have investigated the mechanism underlying the relationships between presence of or search for meaning in life and life satisfaction among Chinese young adults. To reveal these mechanisms, the present study constructed a moderated mediation model to examine the mediating role of perceived stress and moderating role of hope among, thus helping improve life satisfaction among Chinese young adults.

### 1.2 Perceived stress as a mediator

When faced with the same stressor, different individuals often have different cognitions and reactions. The cognitive appraisal theory of stress posits that if an individual believes that the external environment poses a threat to them and they are unable to effectively respond, they have a negative reaction (16). However, if they believe they can overcome the stress, they exhibit a positive reaction (17). The theory provides an integrated framework for explaining the cognitive and reactive processes of individuals facing stressors and has been widely applied in the field of stress research. Perceived stress is a psychological response to a stressful life event after cognitive evaluation of the event (18). When facing a stressful event, young adults who perceive the event as more stressful are more likely to experience less life satisfaction (19). According to the cognitive appraisal theory of stress, when faced with stressors, individuals perform primary and secondary evaluations and adopt corresponding stress-coping styles, thereby affecting their psychological adaptation. As a component of subjective well-being, life satisfaction is considered an indicator of positive adjustment. It is a cognitive evaluation of one’s life (20), which is subjective. When individuals appraise a stressor as a threat to their life, it inevitably affects their evaluation of their quality of life. Therefore, high levels of perceived stress can have a negative effect on life satisfaction. Indeed, Hamarat et al. (21) found that perceived stress is a better predictor of life satisfaction for younger adults. A recent empirical research has also indicated that perceived stress is a significant negative predictor for Chinese emerging adults’ life satisfaction (22).

The meaning-making model states that meaning includes both global and situational meaning. Global meaning refers to an individual’s general orienting system and consists of beliefs, goals, and presence of meaning, whereas situational meaning refers to meaning in the context of a particular environmental encounter (8). According to this model, perceived stress fundamentally arises from an inconsistency between the situational meaning individuals ascribe to stressors and their stable, internalized global meaning. A high level of presence of meaning—serving as a foundational element of global meaning—provides individuals with a clear, stable, and coherent cognitive framework. This framework promotes the appraisal of stressors as manageable challenges rather than fundamental threats, thereby reducing situational-global meaning discrepancies and ultimately lowering perceived stress levels. A study indicated that participants with lower levels of meaning in life reported greater stress than those who reported higher meaning in life (23). A recent study found that presence of meaning in life demonstrates a significant negative predictive effect on perceived stress, whereas search for meaning in life does not (24). Therefore, we speculated that perceived stress may mediate the relationship between presence of meaning in life and life satisfaction among Chinese young adults.

### 1.3 Hope as a moderator

Hope is defined as a cognitive set comprising agency (the belief in one’s capacity to initiate and sustain actions) and pathways (the belief in one’s capacity to generate routes) to reach goals. It is a potential psychological strength that may serve as a protective factor for adolescents facing adverse life events (25). The Strengths theory posits that a psychological strength is a person’s natural capacity to behave, think, or feel in a way that enables their optimal functioning and performance in the pursuit of valued outcomes (25). People who identify and use their strengths were found to report higher levels of subjective and psychological well-being and experience less stress (26). Recent empirical studies have found that hope, as a psychological strength, could significantly positively predict young adults’ life satisfaction after the COVID-19 outbreak (27).

Although a higher presence of meaning can significantly reduce individuals’ perceived stress levels—thereby enhancing life satisfaction—the negative impact of perceived stress on life satisfaction may still vary considerably across different individuals when confronting intractable stressors. In such case, the moderating role of hope as a psychological strength becomes critical. Hope has been found to buffer the negative impact of risk factors on psychological development (28). Moreover, a longitudinal study has provided evidence of the functional role of hope as a moderator in the relationship between stressful life events and adolescent well-being (25). When experiencing perceived stress, individuals with high levels of hope can leverage their strong pathways thinking to conceive multiple effective coping strategies while utilizing agency thinking to sustain effort and confidence in implementation. This psychological strength enables them to more effectively manage stressful experiences, thereby buffering the erosive effect of perceived stress on life satisfaction. Therefore, we speculated that hope may moderate the relationship between perceived stress and life satisfaction among Chinese young adults.

According to the protective factor-protective factor model, when two protective factors impact the dependent variable, they may interact (29). As such, the predictive effect of a protective factor (e.g., meaning in life) on an outcome variable (e.g., life satisfaction) may be moderated by another protective factor (e.g., hope). There are two hypotheses for this interaction: the promotion hypothesis and exclusion hypothesis. The promotion hypothesis refers to one protective factor enhancing the predictive effect of another protective factor on an outcome variable, whereas the exclusion hypothesis refers to one protective factor weakening the predictive effect of another protective factor on an outcome variable (30). For example, hope (a protective factor) can enhance the impact of parent-child communication (another protective factor) on children’s prosocial behavior. Therefore, we hypothesized that the moderating effect of hope in the relationship between presence of meaning in life and life satisfaction may be explained by the promotion hypothesis. Specifically, hope would enhance the effect of presence of meaning in life on life satisfaction of Chinese young adults.

In summary, we proposed the following four hypotheses.

Hypothesis 1: Presence of meaning in life will positively predict life satisfaction among Chinese young adults.

Hypothesis 2: Perceived stress will mediate the relationship between presence of meaning in life and life satisfaction among Chinese young adults.

Hypothesis 3: Hope will moderate the relationship between perceived stress and life satisfaction among Chinese young adults. Specifically, the relationship between perceived stress and life satisfaction will be weaker for Chinese young adults with higher hope.

Hypothesis 4: Hope will moderate the relationship between presence of meaning in life and life satisfaction among Chinese young adults. Specifically, the relationship between presence of meaning in life and life satisfaction will be stronger for Chinese young adults with higher hope.

### 1.4 The Present study

**Figure 1:**
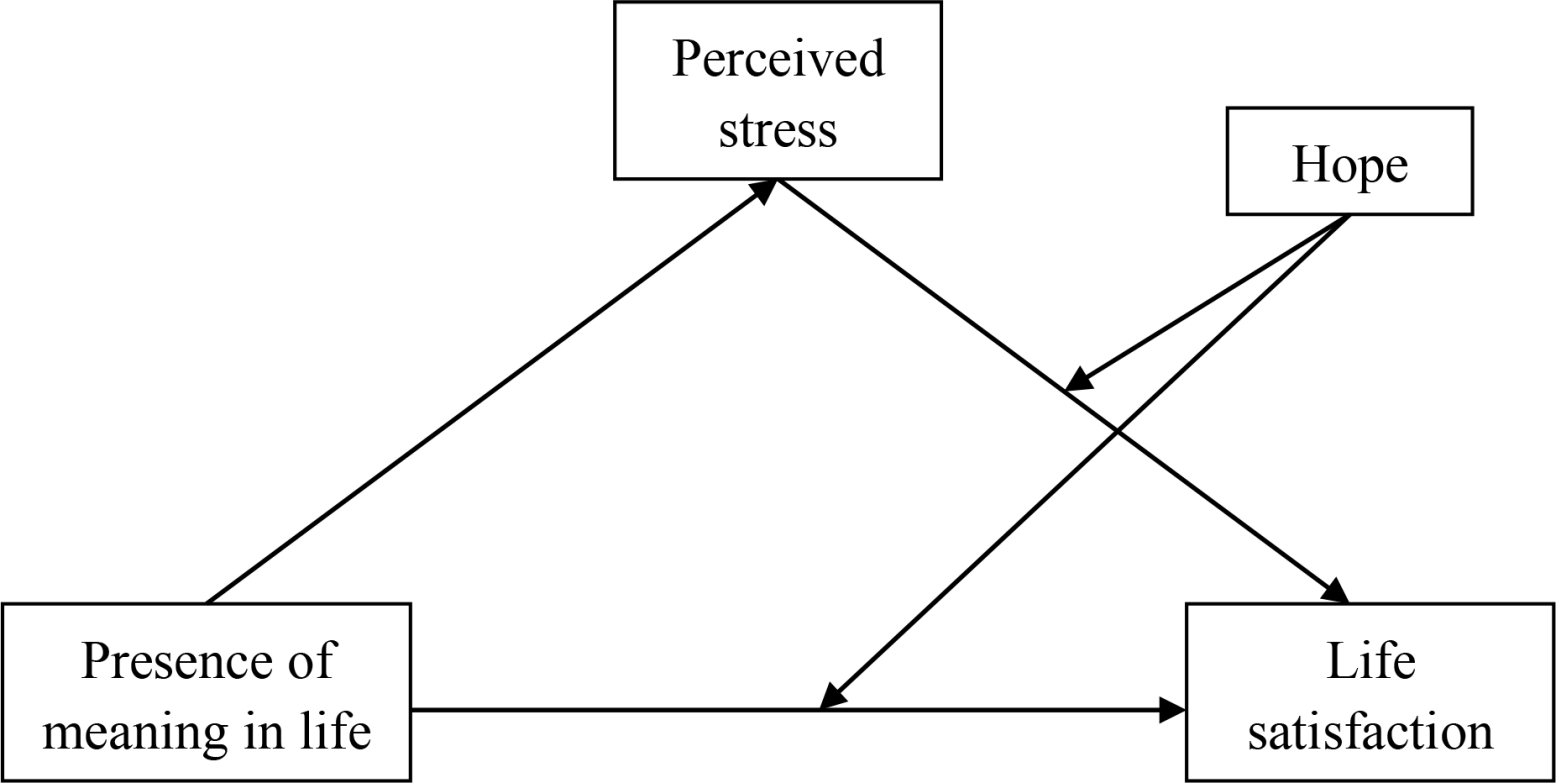
Conceptual model.

This study tested the mechanisms underlying the relationship between presence of meaning in life and life satisfaction among Chinese young adults. we examined a moderated mediation model to answer three questions: (a) Does perceived stress mediate the relationship between presence of meaning in life and life satisfaction among Chinese young adults? (b) Does hope moderate the relationship between presence of meaning in life and life satisfaction? (c) Does hope moderate the mediating effect of perceived stress in the relationship between presence of meaning in life and life satisfaction? (see **Figure 1**).

## 2 Methods

### 2.1 Participants

A total of 909 young adults were selected through stratified random sampling. They were recruited from four large public universities located in Jiangsu Province in China. The mean age was 20.97 years (SD = 1.31) and their ages ranged from 19–22 years. The sample consisted of 333 (36.63%) men and 576 (63.37%) women. A total of 423 (46.53%) participants were from urban families and 486 (53.47%) were from rural families. Regarding the year of study, 237 (26.07%) were freshmen, 246 (27.06%) were sophomores, 219 (24.09%) were juniors, and 207 (22.77%) were seniors.

### 2.2 Measurements

#### 2.2.1 Presence of meaning in life

The Presence of Meaning Subscale of the Chinese version of the Meaning in Life Questionnaire (MLQ) was administered to assess meaning in life among Chinese young adult (31). This subscale has 5 items. Items are responded to using a 7-point scale (1 = never, 7 = always). In this study, Cronbach’s α was 0.78. Confirmatory factor analysis further indicated that the one-factor model demonstrated a good fit to the data (*x^2^/df* = 5.58, *CFI* = 0.91, *TLI* = 0.94, *SRMR* = 0.05, and *RMSEA* = 0.04).

#### 2.2.2 Perceived stress

The Chinese version of the Perceived Stress Scale (CPSS) was used to measure perceived stress (32). This scale has 14 items and two subscales: Perceived Anxiety and Perceived Loss of Control. Items are responded to using a 5-point scale (1 = never, 5 = always). In this study, Cronbach’s α was 0.81 for the CPSS, 0.78 for Perceived Anxiety, and 0.84 for Perceived Loss of Control. Confirmatory factor analysis further indicated that the two-factor model demonstrated a good fit to the data (*x^2^/df* = 5.19, *CFI* = 0.92, *TLI* = 0.95, *SRMR* = 0.05, and *RMSEA* = 0.06).

#### 2.2.3 Hope

The Chinese version of the Hope Scale (CHS) was used to assess hope (33). This scale has 12 items, of which 8 items assess two factors of hope: agency and pathways. The other four items are distracter items. Items are responded to using a 4-point scale (1 = definitely false, 4 = definitely true). Higher scores reflect higher levels of hope. In this study, Cronbach’s α was 0.88 for the CHS, 0.83 for the Agency subscale, and 0.80 for the Pathways subscale. Likewise, confirmatory factor analysis indicated that the two-factor model demonstrated a good fit to the data (*x^2^/df* = 4.72, *CFI* = 0.93, *TLI* = 0.91, *SRMR* = 0.06, and *RMSEA* = 0.06).

#### 2.2.4 Life satisfaction

The Satisfaction with Life Scale (SWLS) was used to measure life satisfaction (34). The SWLS has five items that are responded to using a 7-point scale (1 = strongly disagree, 7 = strongly agree). Higher scores reflect higher life satisfaction. The SWLS demonstrated good reliability and validity in a Chinese sample (35). In this study, Cronbach’s α was 0.85. Likewise, confirmatory factor analysis further indicated that the one-factor model demonstrated a good fit to the data (*x^2^/df* = 4.97, *CFI* = 0.93, *TLI* = 0.95, *SRMR* = 0.04, and *RMSEA* = 0.05).

### 2.3 Procedure

Participants were recruited from four universities located in the two cities of Nanjing and Taizhou, China. The quantitative data were collected by administering a survey questionnaire to the participants in their classrooms at the university during school hours. Each class was provided with two trained psychology graduate students who were responsible for helping students with completing the questionnaire. Participation in the study was completely voluntary and the questionnaire was anonymous.

### 2.4 Statistical analysis

The data were analyzed using SPSS 23.0 and the SPSS macro PROCESS (http://www.afhayes.com) from Hayes (36). First, we calculated descriptive statistics to determine the characteristics of the sample. Second, we performed Pearson’s correlation analysis to evaluate the relationships between the variables, where gender and from an urban or rural area were transformed into dummy variables. Third, we used PROCESS Model 4 and Model 15 to test the moderated mediation model involving perceived stress and hope in the relationship between presence of meaning in life and life satisfaction.

## 3 Results

### 3.1 Common method bias

To ensure the reliability of the research findings, common method bias was assessed following Podsakoff et al.’s (2003) recommendations (37). An unrotated exploratory factor analysis employing Harman’s single-factor test (38) was conducted on all study variables. Results indicated the extraction of 11 factors with eigenvalues exceeding 1.0. The first factor accounted for only 20.85% of the total variance, well below the critical threshold of 40%. These findings indicate no significant common method bias in this study.

### 3.2 Descriptive statistics and correlations

**Table 1** presents the descriptive statistics and correlations among the variables. Grade positively correlated with perceived stress (*p* < 0.05) and negatively correlated with life satisfaction (*p* < 0.001). Being from an urban or rural area was negatively correlated with presence of meaning in life, hope, and life satisfaction (*p* < 0.001), but positively correlated with perceived stress (*p* < 0.05). Presence of meaning in life was positively correlated with hope, and life satisfaction (*p* < 0.001), but negatively correlated with perceived stress (*p* < 0.001). Perceived stress was negatively correlated with hope and life satisfaction (*p* < 0.001), and hope was positively correlated with life satisfaction (*p* < 0.001).

**Table 1:** Descriptive statistics and correlations of main study variables.

Variables	1	2	3	4	5	6	7
1.Gender	1						
2.Grade	0.05	1					
3. From an urban or rural area	0.03	0.04	1				
4. Presence of meaning in life	-0.01	-0.05	-0.20^***^	1			
5. Perceived stress	-0.02	0.08^*^	0.08^*^	-0.56^***^	1		
6. Hope	0.05	-0.09	-0.12^***^	0.59^***^	-0.34^***^	1	
7. Life satisfaction	0.035	-0.14^***^	-0.21^***^	0.51^***^	-0.39^***^	0.66^***^	1
*Mean*	0.36	2.44	0.53	4.58	2.98	2.85	4.02
*SD*	0.48	1.21	0.49	0.97	0.47	0.53	1.28

*N* = 909. Gender: 0, male; 1, female. From urban or rural area: 0, From an urban area; 1, From a rural area.

^*^ *p <*0.05, ^***^ *p <*0.001.

### 3.3 Conceptual model testing

PROCESS Model 4 was used to test the mediating effect of perceived stress in the relationship between presence of meaning in life and life satisfaction. Grade and being from an urban or rural area were controlled for, and the main variables were standardized. The results are presented in **Table 2**. Presence of meaning in life was positively correlated with life satisfaction (*β* = 0.46, *p* < 0.001), which supported for Hypothesis 1. After adding the mediator, the direct relationship was weakened (*β* = 0.37, *p* < 0.01). Presence of meaning in life was negatively associated with perceived stress (*β* = -0.50, *p* < 0.001), and perceived stress was negatively associated with life satisfaction (*β* = -0.17, *p* < 0.001). Consistent with Hypothesis 2, the results of the bootstrapping analyses indicated that perceived stress partially mediated the relationship between presence of meaning in life and life satisfaction (indirect effect = 0.08, *Boot LLCI =* 0.05, *Boot ULCI =* 0.13).

**Table 2:** Regression analysis of the mediating effect of perceived stress.

Regression equation		Overall fitting index			Regression coefficient	
Dependent variable	Independent variable	*R*	*R^2^*	*F*	*β*	*t*
Life satisfaction		0.53	0.28	120.04^***^		
	Grade				-0.10	-3.78^**^
	From an urban or rural area				-0.11	-3.80^**^
	Presence of meaning in life				0.46	16.79^***^
Perceived stress		0.56	0.31	138.42^***^		
	Grade				0.05	1.98^*^
	From an urban and rural area				-0.03	-1.19
	Presence of meaning in life				-0.50	-19.96^***^
Life satisfaction		0.55	0.30	97.56^***^		
	Grade				-0.09	-3.51^**^
	From an urban and rural area				-0.11	-4.03^**^
	Presence of meaning in life				0.37	11.57^***^
	Perceived stress				-0.17	-4.67^***^

^*^ *p <*0.05, ^**^ *p <*0.01, ^***^ *p <*0.001.

PROCESS Model 15 was used to test the moderated mediation model involving perceived stress and hope in the relationship between presence of meaning in life and life satisfaction after controlling for year of study and being from an urban or rural area. The main variables were standardized. The results are presented in **Table 3**. The interaction of perceived stress and hope had a significant effect on life satisfaction (*β* = 0.11, *p* < 0.05). The interaction of presence of meaning in life and hope also had a significant effect on life satisfaction (*β* = 0.14, *p* < 0.01).

**Table 3:** Moderated mediation analysis for presence of meaning in life, perceived stress, hope, and life satisfaction.

Regression equation		Overall fitting index			Regression coefficient	
Dependent variable	Independent variable	*R*	*R^2^*	*F*	*β*	*t*
Life satisfaction		0.70	0.50	126.36^***^		
	Grade				-0.05	-2.45^*^
	From an urban or rural area				-0.09	-4.23^***^
	Presence of meaning in life				0.09	2.71^*^
	Perceived stress				-0.17	-5.58^***^
	Hope				0.63	17.45^***^
	Presence of meaning in life × Hope				0.14	3.47^**^
	Perceived stress × Hope				0.11	2.52^*^
Conditional indirect effect analysis		*β*	*Boot SE*	*Boot LLCI*	*Boot UL CI*	
*M* - *SD*		0.12^***^	0.03	0.072	0.172	
*M* + *SD*		0.04	0.02	-0.013	0.083	
Conditional direct effect analysis		*β*	*Boot SE*	*Boot LLCI*	*Boot UL CI*	
*M - SD*		0.01	0.04	-0.071	0.081	
*M + SD*		0.21^***^	0.05	0.115	0.309	

^*^ *p <*0.05, ^**^ *p <*0.01, ^***^ *p <*0.001.

**Figure 2:**
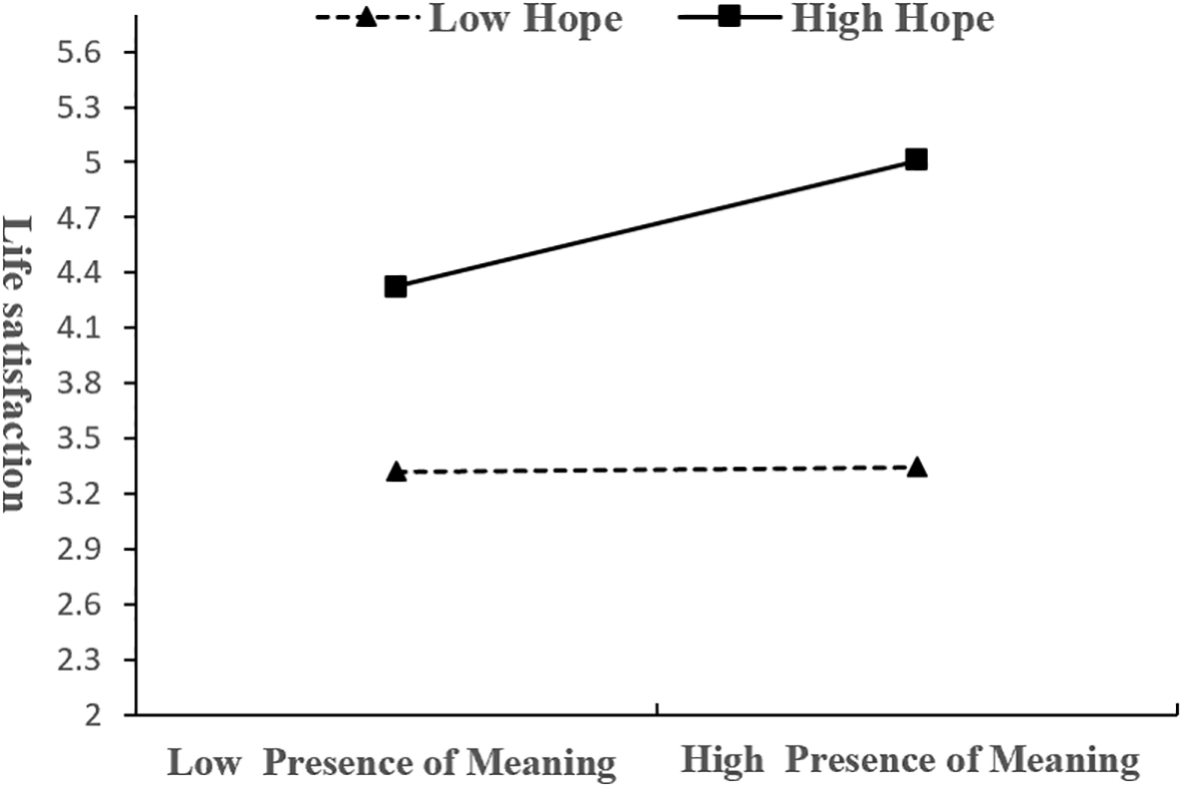
Moderating effect of hope on the relationship between the presence of life meaning and life satisfaction.

**Figure 3:**
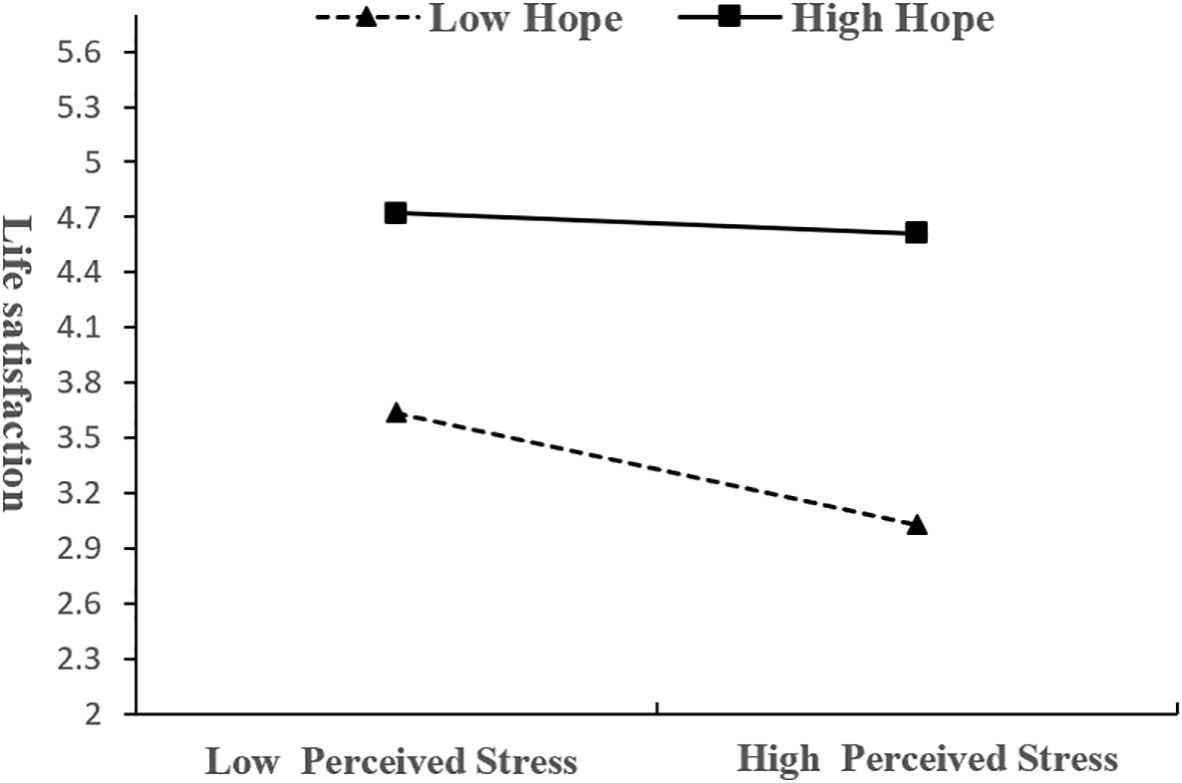
Moderating effect of hope on the relationship between perceived stress and life satisfaction.

Simple slopes tests were used to interpret the significant interactions, and hope scores were dichotomized as low (*M* - *SD*) or high (*M* + *SD*). As shown in **Figure 2**, the predictive effect of presence of meaning in life on life satisfaction was significantly stronger for individuals with high hope (*β* = 0.35, *t* = 5.22, *p* < 0.001) than for those with low hope (*β* = 0.01, *t* = 0.23, *p* = 0.82), indicating that increase in hope enhanced the effect of presence of meaning in life on life satisfaction. Meanwhile, the predictive effect of perceived stress on life satisfaction was weaker for individuals with high hope (*β* = -0.11, *t* = -0.95, *p* = 0.33), and the predictive effect of perceived stress on life satisfaction was stronger for individuals with low hope (*β* = -0.63, *t* = -6.03, *p* < 0.001), indicating that increase in hope weakened the effect of perceived stress on life satisfaction (**Figure 3**). The effect of presence of meaning in life on life satisfaction and the 95% bootstrap values are shown in **Table 3**. The findings demonstrated that the mediating role of perceived stress in the relationship between presence of meaning in life and life satisfaction was moderated by hope. Specifically, perceived stress fully accounted for this relationship at lower levels of hope (*β*= 0.12, *95%CI* [0.072, 0.172]), whereas this mediating pathway was nonsignificant at elevated hope levels (*β*= 0.04, *95%CI* [-0.013, 0.083]). Thus, Hypotheses 3 and 4 were supported.

## 4 Discussion

The present study constructed a moderated mediation model to examine the mechanisms underlying the relationship between the presence of meaning in life and life satisfaction among Chinese young adults. The results indicated that perceived stress played a mediating role in the association between the presence of meaning in life and life satisfaction, and this mediated relationship was moderated by hope.

### 4.1 Presence of meaning in life and life satisfaction among Chinese young adults

Building upon prior research (13), we investigated presence of meaning in life as separate constructs and explored its relationships with life satisfaction. The results showed that presence of meaning in life positively predicted life satisfaction among Chinese young adults, which is consistent with the findings of previous studies (13, 15). Presence of meaning in life is a cognitive dimension that provides people with an overall framework for understanding themselves, the world, and the relationship between themselves and the world (39). When people have a meaning in life, they make more positive and meaningful cognitive evaluations of their lives and adopt positive coping strategies to cope with the stressors (40), which ultimately contributes to their satisfaction with life.

### 4.2 The mediating role of perceived stress

The findings suggest that perceived stress mediates the association between the presence of meaning in life and life satisfaction among Chinese young adults. Specifically, young adults who reported higher levels of meaning in life demonstrated lower perceived stress, which in turn was associated with greater life satisfaction. This can be attributed to the tendency of individuals with established life meaning to engage in more positive cognitive appraisals of life circumstances and employ adaptive coping strategies when confronting stressors (40). In line with the cognitive appraisal theory of stress, individuals initially conduct primary appraisal (assessing threat significance) followed by secondary appraisal (evaluating coping resources), ultimately determining their stress-coping approaches and subsequent psychological adaptation outcomes (16, 17). During the primary appraisal stage, individuals with a higher presence of meaning in life demonstrate a greater tendency to perceive stressors as opportunities for fulfilling life goals and actualizing personal values, rather than mere threats. This positive primary appraisal significantly attenuates perceived threat severity, thereby reducing perceived stress. At the secondary appraisal stage, presence of meaning provides robust psychological resources and coping efficacy. When confronting stressors, young adults with high levels of presence of meaning actively mobilize personal resources and employ diversified adaptive coping strategies, empowered by the conviction and motivation derived from their presence of meaning. These effective strategies facilitate practical problem-solving and diminish perceived stress levels. As perceived stress decreases, negative emotions subside while positive affect and psychological resources are restored and enhanced. This process fosters more favorable evaluations across life domains, ultimately elevating life satisfaction.

### 4.3 The moderating role of hope

The present study also indicated that hope not only moderated the relationship between presence of meaning in life and life satisfaction, but also the mediating effect of perceived stress in the relationship.

Firstly, the effect of presence of meaning in life on life satisfaction strengthened as hope increased. This result confirms the promotion hypothesis of the protective factor-protective factor model. Snyder et al. (41) pointed out that individuals with high levels of hope have clear future goals and can adopt various methods to overcome the obstacles in the process of achieving goals. For individuals with high levels of hope, presence of meaning can increase positive and constructive behaviors, thereby enhancing their sense of value and improving their level of life satisfaction. Conversely, young adults with diminished hope levels exhibit attenuated benefits from presence of meaning in life, likely due to deficient life goal articulation and limited utilization of adaptive problem-solving approaches when confronting challenges. The study have further shown that, in the long run, life satisfaction is deeply influenced by individual life goals (42). The absence of life goals might undermine the directional focus of proactive behaviors and impair problem-solving efficacy, thereby disrupting the effective translation of presence of meaning into sustained psychological benefits and ultimately constraining the enhancement of life satisfaction. Therefore, for young adults with low levels of hope, the effect of presence of meaning in life on life satisfaction was weaker.

Secondly, our study found that hope moderates the effect of perceived stress on life satisfaction, indicating that the effect of perceived stress weakened as hope increased. This result validates the protective factor model (43), which means that as a protective factor for young adults, hope could effectively buffer the adverse effects of risk factors such as perceived stress on their mental health and further promote their resilience. Hope refers to the strength of individuals to face the future (44). Individuals with high levels of hope tend to focus more on achieving future goals rather than on their current state. Even if they encounter various pressures while pursuing goals, they would actively take measures to minimize them and successfully achieve their goals. Therefore, for young adults with high levels of hope, perceived stress has less impact on their life satisfaction. Thus, hope may buffer the effects of perceived stress on life satisfaction.

Moreover, our study found that perceived stress exhibit a complete mediating effect in the relationship between presence of meaning in life and life satisfaction for Chinese young adults with low hope levels, but not for those with high hope levels. According to Snyder’s hope theory, young adults with low hope levels often fail to translate abstract meaning in life into specific behavioral strategies when facing stressful events due to the lack of pathways thinking. This inability promotes the elevation of perceived stress, which reduces their life satisfaction. In this case, presence of meaning in life remains confined to the conceptual level, unable to directly enhance life satisfaction due to its failure to drive substantive behaviors that would otherwise improve satisfaction. Therefore, for Chinese young adults with low hope levels, perceived stress exhibits a complete mediating effect in the relationship between presence of meaning and life satisfaction. Conversely, young adults with high hope levels can transform presence of meaning in life into operational coping strategies through pathways thinking and agency thinking. This transformation process allows presence of meaning to act directly on life satisfaction bypassing the mediation of perceived stress. In other words, for these young adults, presence of meaning in life itself becomes a tool to cope with stressful events, rather than indirectly enhancing life satisfaction by reducing perceived stress. Therefore, for Chinese young adults with high hope levels, perceived stress does not exhibit a significant mediating effect in the relationship between presence of meaning in life and life satisfaction.

### 4.4 Implications

To our knowledge, our study is the first to focus on the underlying influence of perceived stress and hope in the association between presence of meaning in life and life satisfaction among Chinese young adults in the context of the meaning-making model and the strengths theory. This study provides a new perspective on the relationship between presence of meaning in life and life satisfaction among Chinese young adults. Another contribution of this study is in expanding our understanding of well-being and resilience among non-Western young populations. The present research found that, in the context of Chinese culture, hope, as a protective factor, could enhance the impact of presence of meaning in life (another protective factor) on life satisfaction among young adults and effectively buffer the adverse effects of risk factors such as perceived stress on their life satisfaction. Moreover, the results can be used to guide future interventions aimed at improving life satisfaction among Chinese young adults. Firstly, educators can increase the levels of presence of meaning in life to improve life satisfaction among young adults through various intervention strategies, such as Satir model group intervention. According to a recent study, Satir model group intervention can significantly improve the levels of presence of meaning in life among Chinese young adults (45). Secondly, we can enhance life satisfaction among Chinese young adults by cultivating their hope through targeted interventions. Substantial empirical evidence demonstrates that young adults’ hope levels can be significantly elevated via interventions such as group counseling programs (46, 47).

### 4.5 Limitations

Several limitations of the present study need to be highlighted. First, the sample was derived from four large public universities located in Jiangsu Province, China, which may not represent all young adults. Therefore, future studies should expand the sample. Second, this study employed a cross-sectional design, which precludes definitive causal inferences between the variables. Future research may employ a three-wave longitudinal tracking design and apply cross-lagged panel models to establish causal relationships. Third, self-report methods were used for data collection, which can be affected by social desirability bias and recall bias (48). Future investigations should adopt multimodal data collection approaches, integrating cross-channel data such as physiological indicators, behavioral observations, and multi-source reports to establish methodological triangulation.

## 5 Conclusions

In this study, we investigated the roles of perceived stress and hope in the relationship between presence of meaning in life and life satisfaction among Chinese young adults. The results indicated that presence of meaning in life was positively associated with life satisfaction among Chinese young adults. Furthermore, perceived stress played a mediating role in the association between presence of meaning in life and life satisfaction, and this mediating effect was moderated by hope. Overall, the results of this study provide a new perspective for exploring the relationship between presence of meaning in life and life satisfaction among Chinese young adults. Interventions to enhance presence of meaning in life and hope, as well as reduce perceived stress, are potential strategies to help increase life satisfaction among Chinese young adults.

## Data Availability

The datasets presented in this article are not readily available because The data that support the findings of this study are available on reasonable request. The data are not publicly available due to information that could compromise the privacy of research participants. Requests to access the datasets should be directed to Yan Zhang, peterzhang1108@163.com.
